# Helminth parasite body size scaling with host species size

**DOI:** 10.1098/rspb.2026.1330

**Published:** 2026-10-14

**Authors:** Tad Dallas, Lauren A. Holian, Grant Foster

**Affiliations:** ^1^ Department of Biological Sciences, University of South Carolina System, Columbia, SC, 29208, USA; ^2^ Center for the Ecology of Infectious Diseases, University of Georgia, Athens, GA, 30602, USA; ^3^ Department of Biology, Texas Wesleyan University, Fort Worth, TX 76105, USA

**Keywords:** parasite body size, parasite macroecology, Harrison’s rule, body size scaling

## Abstract

Host and parasite body size frequently scale positively (often referred to as *Harrison’s rule*), potentially as a result of fundamental constraints on large parasite species simply being morphologically incapable of infecting small host species, or through a variety of alternative proposed mechanisms. Regardless of mechanism, Harrison’s rule does have some empirical support. Here, we leverage several trait databases and a large set of host–helminth parasite interactions to gauge support for Harrison’s rule in parasitic helminths. We fit generalized additive models from both the host perspective (do larger host species tend to have larger average parasites?) and the parasite perspective (do larger parasite species tend to infect larger average host species?) across a range of host taxa including mammals, birds and fish. We find support for both relationships in mammals, moderate support in fish and very limited support in birds, relative to a null model. Together, we demonstrate positive host–parasite body size scaling in parasitic helminths, suggesting the potential for fundamental morphological constraints on host–parasite interactions, while also highlighting the clear differences in body size scaling across host taxa.

## 1. Introduction

The availability of data and the conceptual development of large-scale ecological theory—what some would consider ‘macroecological laws’—has created an opportunity to explore high-level scaling relationships in host–parasite associations [1,2]. This includes explorations of latitudinal patterns of parasite diversity [3] and body size [4], large-scale spatial patterns of host use and species roles in host–parasite networks [5] and biogeographical relationships such as species-area relationships in host and parasite communities [6]. While many of these scaling relationships originated in biogeography and macroecology, scaling rules have been developed specific to host–parasite associations as well.

One such relationship is Harrison’s rule, describing the tendency for large parasites to be found on larger-bodied host species [7,8]. This was originally described for lice (*Rallicola* species) on a small number of bird species (*Apteryx* species). Evidence for this relationship largely comes from ectoparasites [9,10,11], though internal macroparasites have also provided support [8,12]. This relationship is not always found [13], and there is no consensus on the underlying mechanism [1,14]. The rationale is that larger parasites may be unable to infect smaller host species, or come at too high of a cost in terms of damage to host tissues, leading to a tendency for larger parasites to infect larger host species. Meanwhile, smaller parasites may not have this constraint, putatively allowing smaller parasites to infect both small and large host species (there is no relationship between parasite body size and host range; see supplementary material).

Regardless of whether the host–parasite body size scaling relationship is linear or wedge-shaped, there is important nuance to how the relationship is explored. The relationship can be explored by considering all host–parasite associations, potentially over-representing parasites with large host range or host species with higher parasite species richness, or by considering summary statistics (e.g. mean or maximum body size) for either host or parasite. That is, the relationship could be explored from the host perspective (larger host species tend to be infected by larger average parasite species) or the parasite perspective (larger parasite species tend to infect larger average host species). Finally, the appropriate estimate of size itself is non-trivial, as one-dimensional measures of morphology such as body length [15,16] are commonly used, though efforts have been made to approximate parasite volume [17,18].

Here, we use two existing databases on host and helminth parasite body sizes across mammal, bird and fish hosts to explore host–parasite body size scaling relationships from both host and parasite perspectives, considering parasite length, width and biovolume. We compare observed relationships with a null model that preserves the number of species interactions but shuffles them to remove any coevolutionary or morphological constraint on the interaction. We find positive body size scaling relationships from both host and helminth parasite perspectives that are stronger than what would be expected under our null model for mammals and fish, but not for birds. Further, we did not find consistent body size scaling between models trained from the host perspective compared with the parasite perspective within the same host taxonomic group. Together, we provide support for the existence of morphological constraints on host–parasite interactions, highlighting the fundamental constraints on parasite specificity and host range.

## 2. Methods

### (a) Helminth life cycle database

We used the helminth life cycle database compiled by Benesh *et al.* [19], which consists of over 10 000 records across 973 parasite species. These data contain morphological measurements for multiple stages of many complex life cycle helminth parasites. These data were processed to only include adult helminth parasites (but see supplementary material for an exploration of Harrison’s rule in larval parasites). These data encompass a broad phylogenetic range of host species. To explore a range of taxonomic groups, we used existing trait databases on mammals (COMBINE [20]), birds (AvoNet [21]) and fish (fishBase [22]) to obtain body size measures (measured as adult body mass). Host body mass may not actually capture some of the proposed underlying mechanisms that would allow larger hosts to be infected by larger parasites. That is, we make the assumption that host size is a good surrogate for the size of the habitat available to the helminth parasites, as one proposed mechanism is that within-host size constraints prevent large parasites from infecting smaller host species. It is important to note that helminth parasites can infect a diversity of tissues and are potentially found throughout the body of the host instead of solely in the host intestine.

### (b) Parasite body size measures

For helminth parasite species, we consider parasite length and width, as compiled by Benesh *et al.* [19]. These include host species-specific estimates of helminth size from the primary literature, allowing for some intraspecific variation for the same parasite species. Across records, we considered the mean length and width for each parasite, as we were focused primarily on across-species variation in body size and not intraspecific variation or variation in helminth body size across host species. Further, on average there were between three and four records of length and width for each adult helminth parasite species, which may not be enough to assess intraspecific body size variation as a function of host species infected, especially given the potential for environmental constraints on parasite size.

Length and width are common measures of parasite body size [16,23], but may not fully capture aspects of body size that may limit parasite specificity. Parasite biovolume is a measure that incorporates length and width [17,18]. As a coarse estimate of parasite biovolume, we assume a cylindrical helminth and calculate helminth volume as a function of parasite length and width. It is important to note that parasite body size and/or biovolume are not necessarily proportional to the resulting impact on host fitness [8]. Smaller parasites may have life histories allowing them to multiply faster, such that *per capita* effect is low while the true impact to host fitness may be much larger. Ideally, experimental infections linking parasite body size to host fitness impacts would allow an understanding of the scaling between parasite body size and resulting impact on the host.

### (c) Assessment of Harrison’s rule

Harrison’s rule asserts that parasite body size should increase as host body size increases [7], which can be assessed in a number of ways. First, we could include all data on host and parasite body sizes, but this inherently biases any regression analysis towards hosts with many parasites or parasites with wide host range, as the unit of analysis would be the pair of body sizes between host and parasite. To avoid this, we considered two different scenarios. First, as host species body size increases, the average parasite body size will increase. Second, as parasite body size increases, the average host body size will increase. This reduces the unit of analysis to either the host or the parasite, respectively (figure 1). We fitted generalized additive models (GAMs) to both of the scenarios described above, following thin plate regression splines and basis dimension (k) set to 10. This resulted in a total of six models per taxonomic group (mammals, birds and fish), as parasite body size was estimated as length, width and biovolume. These models have the potential to overfit, resulting in large estimated degrees of freedom (e.d.f.) values and inflated pseudo-$R^{2}$ values. We explore this in the supplementary material by dividing the data into a train and a test set and exploring the distribution of $R^{2}$ on the test set not used to train the model. We also include Spearman’s rank correlation coefficients to demonstrate that the GAMs and simple correlation tend to arrive at the same conclusion (supplementary material).

**Figure 1.**
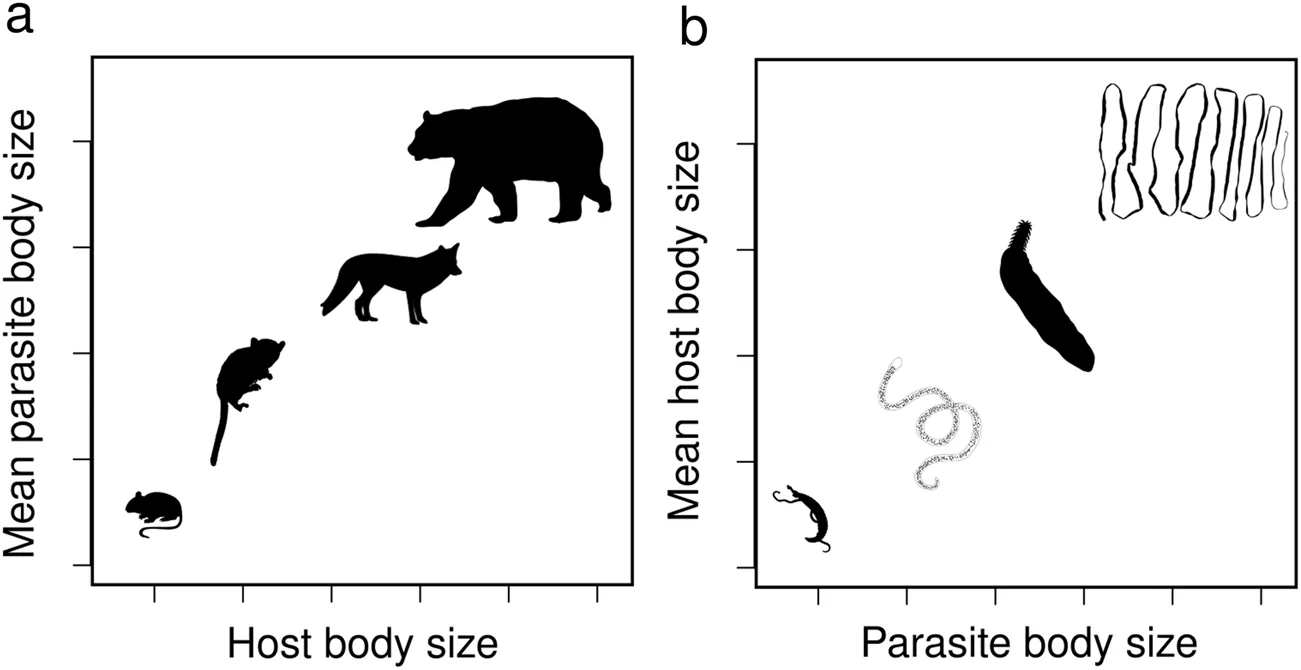
Harrison’s rule can be viewed through two lenses at the species level. (a) Host species body size may positively scale with average parasite size (the host perspective), or (b) parasite body size may be positively related to average host body size (the parasite perspective).

### (d) The role of phylogenetic information

Accounting for host and parasite phylogenetic information when dealing with host–parasite interaction data [24,25] and associated traits [26,27] can yield valuable insight into the underlying drivers of host–parasite associations [28,29]. As it applies to Harrison’s rule, the hypothesis then tests the independent influence of host or parasite body size after accounting for the potential phylogenetic signal of body size. This makes the assumption that body size has a phylogenetic signal, which we see in the bird data but not for mammals or fish (supplementary material). The decision to fit GAMs was to allow for nonlinear relationships between host and parasite body size. We incorporated host phylogenetic information into these models and found that the estimated degrees of freedom—which can be interpreted as a measure of the degree of this nonlinearity—for this effect to be quite high. This led to over-inflated $R^{2}$-values and likely seriously overfitted models. We have presented these models in the supplementary material and treat models that do not incorporate host phylogenetic information here. While the fraction of host species that had to be removed because we had no phylogenetic information given the tree was low for host species (generally less than 10% of species), this would have removed over 95% of helminth parasite species from our data.

### (e) A null model of host–parasite body size scaling

Depending on the distribution of body sizes, resulting scaling relationships may simply be a function of host range or parasite species richness, combined with the underlying body size distribution. To explore this, we assembled random host ranges or parasite communities constrained by the observed interactions. For every host species, we sampled parasite species randomly, but kept parasite species richness for that host species constant. Similarly, for each parasite species, we randomly sampled host body sizes constrained by observed parasite host range. This process was done 100 times for each host and parasite species to generate a null expectation for the relationship between host and parasite body sizes from both host perspective and parasite perspective. All analyses were performed in R [30].

## 3. Results

### (a) Harrison’s rule from the host perspective

Larger-bodied host species tended to be infected by helminth species with larger average body sizes (figure 2). This is true whether parasite body size is considered as length, as width or as biovolume (assuming a cylindrical parasite). Fitted GAMS explained variable amounts of the variation in the data for width and length, but generally explained more variation in mean parasite biovolume (table 1). Models fitted to the null data were not significant (see supplementary material for model fits), or when the model did have a significant term, it explained functionally zero variation in the data (supplementary material, table S8). Clear differences across host taxa existed, with GAM model fits to mammals tending to explain more variation, followed by fish, and only a small amount of variation on average for bird host body size scaling relationships (table 1 and figure 2). This serves to highlight potential taxonomic differences in the strength of host–parasite body size scaling relationships or the influence of the range of body sizes present in the data.

**Figure 2.**
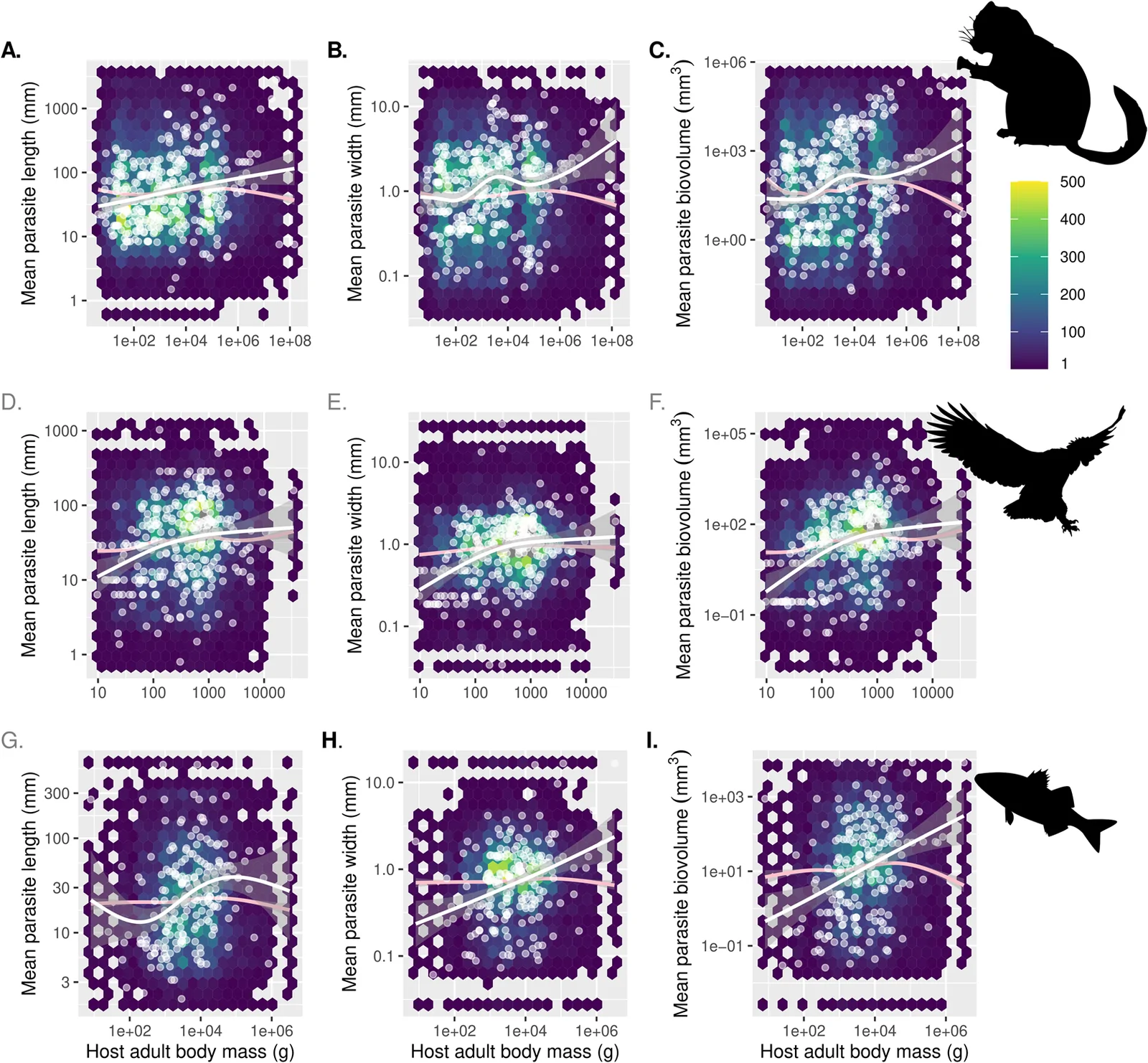
Evidence for Harrison’s rule from the host perspective. Host body size was positively related to mean parasite length (A,D,G), mean parasite width (B,E,H) and mean parasite biovolume (C,F,I). White points and lines correspond to the empirical data and fits from generalized additive models, while background colour gradient and pink lines correspond to the underlying null distribution and generalized additive model fit to null data. Bold panel labels correspond to significant model fits.

**Table 1.** Fitted generalized additive models for Harrison’s rule for both host (top) and helminth parasite (bottom) perspectives provide support for positive host–parasite body size scaling relationships. d.f., degrees of freedom used for F-test; e.d.f., estimated complexity of the smoothed term; adj. $R^{2}$, adjusted variance explained. The s() notation refers to the smoothed effect of the given predictor variable. d.f. is degrees of freedom used for *F*-test, e.d.f. is the estimated complexity of the smoothed term, and adj. R^2^ is the adjusted variance explained. Numbers in bold type correspond to significant model fits.

	response	predictor	e.d.f.	d.f.	*F*	p-value	adj. $R^{2}$
mammals	mean parasite width	s(host mass)	5.09	5.88	5.08	**<0.001**	0.07
	mean parasite length	s(host mass)	8.09	8.66	15.66	**<0.001**	0.26
	mean parasite biovolume	s(host mass)	8.71	8.96	61.26	**<0.001**	0.60
birds	mean parasite width	s(host mass)	1.00	1.00	0.43	0.51	0.00
	mean parasite length	s(host mass)	3.80	4.47	1.26	0.35	0.01
	mean parasite biovolume	s(host mass)	1.00	1.00	0.01	0.94	0.00
fish	mean parasite width	s(host mass)	8.65	8.96	14.78	**<0.001**	0.38
	mean parasite length	s(host mass)	1.00	1.00	0.03	0.85	0.00
	mean parasite biovolume	s(host mass)	8.31	8.85	6.77	**<0.001**	0.21
mammals	mean host size	s(width)	6.87	7.93	2.46	**0.01**	0.05
	mean host size	s(length)	7.03	8.10	4.51	**<0.001**	0.09
	mean host size	s(biovolume)	8.26	8.79	23.34	**<0.001**	0.38
birds	mean host size	s(width)	2.06	2.52	1.31	0.39	0.01
	mean host size	s(length)	6.25	7.28	1.51	0.16	0.03
	mean host size	s(biovolume)	8.04	8.69	6.35	**<0.001**	0.17
fish	mean host size	s(width)	8.76	8.98	17.90	**<0.001**	0.40
	mean host size	s(length)	1.00	1.00	0.02	0.90	0.00
	mean host size	s(biovolume)	8.45	8.84	5.56	**<0.001**	0.15

### (b) Harrison’s rule from the parasite perspective

Larger-bodied helminth parasites tended to have host ranges with larger average host body mass (figure 3). Fitted GAMs explained less variation than from the host perspective (table 1) across all host taxa, despite models being fitteed to more data. That is, since the unit of analysis is either host or parasite species, fitted models tended to have far fewer host than parasite species (e.g. 387 mammal host species and 878 mammal helminth species). Despite this, models relating smoothed helminth biovolume to average mammal ($R^{2}$ = 0.38), bird ($R^{2}$ = 0.17) and fish ($R^{2}$ = 0.15) host size explained an appreciable amount of variation in the data. We found modest support that larger bird helminths tended to infect larger bird species in terms of biovolume (table 1), and that larger fish helminths—in terms of both width ($R^{2}$ = 0.40) and biovolume ($R^{2}$ = 0.15)—tended to infect larger fish species.

**Figure 3.**
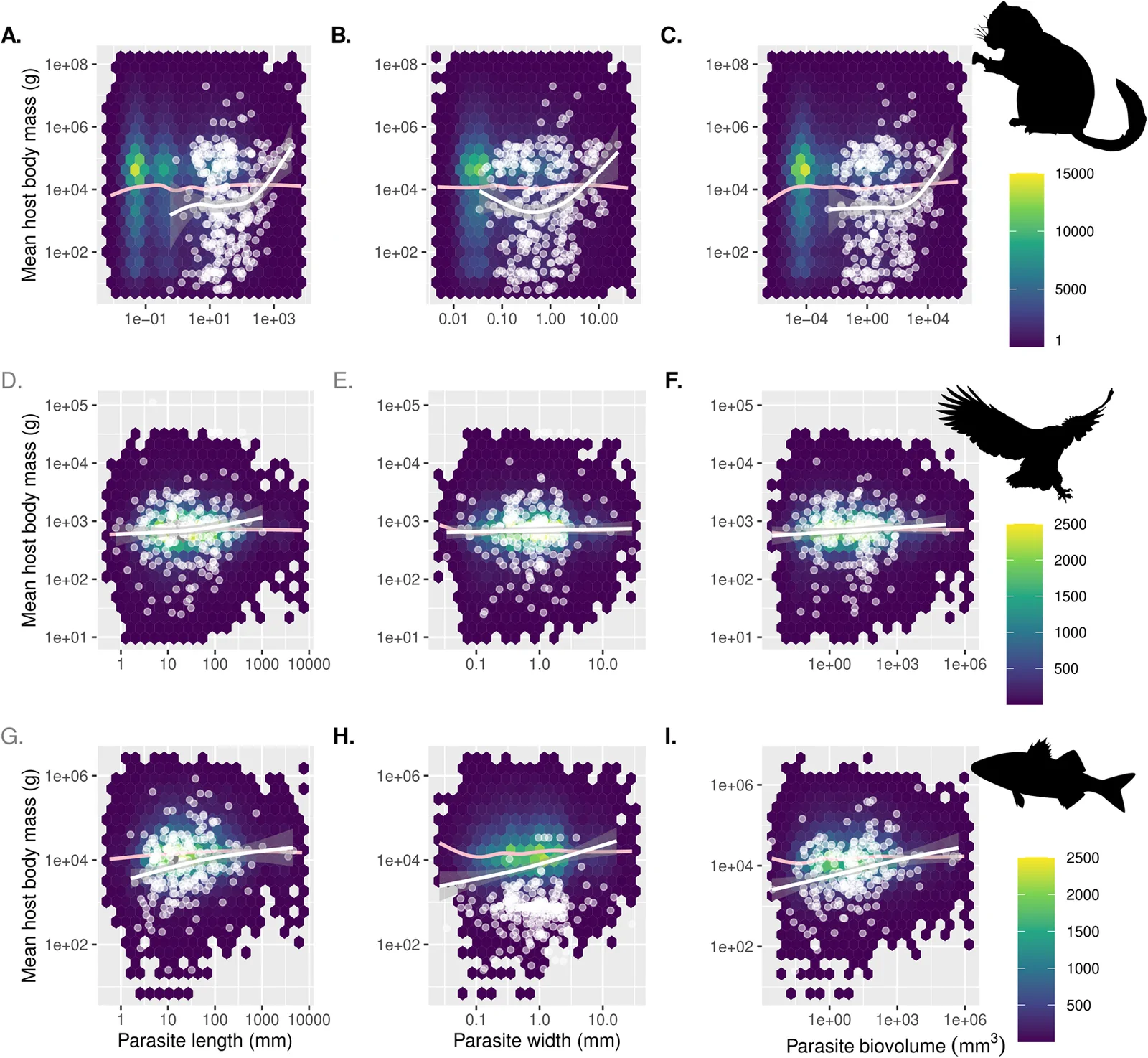
Evidence for Harrison’s rule from the parasite perspective. Parasite length (A,D,G), width (B,E,H) and biovolume (C,F,I) were related to average host body size. White points and lines correspond to the empirical data and fits from generalized additive models, while background colour gradient and pink lines correspond to the underlying null distribution and generalized additive model fit to null data. Bold panel labels correspond to significant model fits.

## 4. Discussion

We generally found positive scaling relationships between host and helminth body sizes from both host and parasite perspectives, but the strength and detectability of these relationships varied across taxa. At least for mammals and fish, larger host species were infected by larger average parasite species. Similarly, in all taxa (mammals, birds and fish), larger parasite species generally infected larger average host species. These results were not statistical artefacts, as evidenced by our null model, and were also not a simple result of larger host species having higher parasite species richness (supplementary material). Fitted models explained substantial variation for some host taxa, especially when parasite size was estimated as biovolume. Together, we leveraged nonlinear models and the use of null model randomizations to explore support for Harrison’s rule in helminth parasites from both host and parasite perspectives, finding clear evidence of positive body size scaling relationships.

Aspects of host morphology inherently constrain parasite morphology, as observed with lice parasitizing diving hosts, where rounder lice were found to parasitize host species that dived deeper [31]. Further, previous studies have found a relationship between louse head size and the width of avian host feather hair shaft, providing another morphological constraint on the physical interaction between host and parasite [13]. However, clearly there are different constraints on helminth parasite body size compared with louse body size. So while it is possible that these morphological constraints exist for helminth parasites, it is perhaps more likely other processes determine parasite body size. That is, parasite competition within infected host species, host–parasite coevolutionary processes and host diet breadth may be more important in determining parasite community assemblages and parasite body sizes within infected host species. The role of space limitation and within-host competition might lead to a constraint only on the maximum parasite size. We do find the same body size scaling when considering maximum parasite body size (supplementary material, figure S1 and table S9), but fitted models explained considerably less variation relative to the models fitted on mean parasite body size.

There was not necessarily a clear link between host and parasite perspective models for a given relationship (e.g. the bird parasite biovolume model had an $R^{2}$ of 0.17 from the helminth perspective and an $R^{2}$ of 0 from the host perspective). We also found host groups differing in their scaling relationships, where groups with a wider range of host body size tended to show more support for body size scaling relationships. That is, the strongest support for Harrison’s rule comes from mammals (which had a maximum size on the order of 100000 kg), slightly weaker support for fish (which had a maximum size on the order of 1000 kg) and little support for birds (which had a maximum size on the order of 10 kg). It is possible that the range of body sizes inherently constrained the ability to detect positive body size scaling relationships (i.e. the range of body sizes for mammals was so much larger than the range of body sizes for birds or fish). This could point to the importance of coevolution, host traits (other than body size) and other ecologically relevant factors in constraining host–parasite interactions at smaller scales (e.g. within particular taxonomic groups without a wide range of body sizes).

It is also possible that the average parasite size increases simply as a function of the relationship between host body size and parasite species richness. That is, larger host species may have more parasite species infecting them, which has the potential to artificially inflate the average parasite body size. We addressed this in two different ways. First, the use of the null model starts to address this. Second, we fitted GAMs to mean parasite body size as a function of parasite species richness (see supplementary material). We observed a positive relationship between parasite species richness and mean parasite length, width and biovolume. However, these positive relationships were no different from expected under a null model of randomly sampling parasite species that could infect a hypothetical host species. This suggests that host species that are infected by more helminth parasites may have larger average parasites, but it is likely a statistical artefact and not a property of the host species that leads to more parasitized host species also having larger average parasites.

The data we used represent the current best large-scale body size data on mammal, bird and fish hosts and their respective helminths. However, we cannothet necessarily trust model fits if there are inherent biases or issues with the underlying data. A common criticism of large-scale explorations is that sampling and publication biases are present, limiting confidence in the data’s ability to address some scientific questions. These biases are insidious in that they are almost always present and almost never easy to get around. Considering the mean parasite body size at the species level starts to get around some issues with small sample sizes or geographical biases in infection patterns, and the underlying null model behind each analysis may also aid in reducing the effect of these biases. Investment in the production and curation of these data sources is important to refine our knowledge of the fundamental constraints of host–parasite interactions.

Altogether, we find support for Harrison’s rule in parasitic helminths, where there is a positive relationship between (i) parasite body size and average host size (parasite perspective) and (ii) host body size and average helminth parasite size (host perspective). This suggests that there are fundamental constraints on helminth infections of host species, highlighting the potential for host and parasite body size data to be incorporated into host–parasite association prediction efforts [32]. Further exploration of high-level scaling patterns in parasites has the potential to inform more than link prediction efforts. Applications of macroecological theory to parasite species and communities offer the potential to refine existing theory and disentangle support for proposed mechanisms [3].

## Supplementary Material



## Data Availability

Data and R code are available on figshare at [33]. Cite original data sources for trait [19–21] and phylogenetic [34–36] data. Supplementary material is available online [37].
