# Supplementary material for: Helminth parasite body size scaling with host species size
Source: Proc Biol Sci. 2026 Oct 14;293(2080):20261330. doi: 10.1098/rspb.2026.1330 (PMC13678628; doi:10.1098/rspb.2026.1330)
Supplement: Supplementary file 1 [file rspb.2026.1330.zip › rspb.2026.1330/rspb20261330_si_001.pdf]

Supplemental information for: Helminth parasite body size scaling with host species size

Tad A Dallas[a,b,*], Lauren Holian[a] and Grant Foster[a]

[a] *Department of Biology, University of South Carolina, Columbia, SC, 29205, USA*
[b] *Center for the Ecology of Infectious Diseases, University of Georgia, Athens, GA, 30602*

*Corresponding author: tad.a.dallas@gmail.com

The role of phylogenetic information

Controlling for phylogenetic relatedness is important to understanding if co-evolutionary relationships could lead to pattens such as body size scaling relationships independent of any *true* effect of body size. The underlying idea would be that if there was a phylogenetic signal in host body size, and parasites showed phylogenetic specificity (infecting a non-random group of closely related host species), Harrison's rule could be observed simply as a function of these factors and not driven by the traditional explanations of morphological constraints on infection processes or evolving specificity to maximize parasite reproductive output (i.e., a larger parasite may be able to infect a smaller host species, but may be able to produce more offspring in larger host species). We did not observe a phylogenetic signal in either host body size (Table 2) or mean parasite size (Table 1).

We used phylogenetic data from published studies, including the a mammal tree from Vertlife [1], fish tree from Tree of Life [2], bird tree from [3], and helminth parasite tree from [4]. We first explored the existence of a phylogenetic signal across host groups in any of the parasite body size measures we considered.

Table 1: We observed limited evidence for a phylogenetic signal (K) in mean parasite body size across our host taxa. p-values are based on 1000 simulations, and significant p-values are bolded.

Host taxa	Trait	K	p-value
Mammals	Mean parasite length	0.044	0.213
	Mean parasite width	0.028	0.608
	Mean parasite biovolume	0.045	0.317
Fish	Mean parasite length	0.186	**0.028**
	Mean parasite width	0.151	0.068
	Mean parasite biovolume	0.094	0.294
Birds	Mean parasite length	0.346	**0.007**
	Mean parasite width	0.513	**0.007**
	Mean parasite biovolume	0.414	0.158

Table 2: We observed limited evidence for a phylogenetic signal (K) in host body size across our host taxa. p-values are based on 1000 simulations, and significant p-values are bolded.

Host taxa	K	p
Mammals	0.039	0.592
Birds	13.702	0.001
Fish	0.101	0.476

We find limited evidence that average parasite body size had any sort of signal across the host phylogenies. Another underlying assumption would be that host body size is phylogenetically conserved, such that parasites infecting phylogenetically similar host species could inadvertently 'specialize' on a subset of host body sizes. However, we only see a significant phylogenetic signal in host bod size in birds (Table 2).

For completeness, we still incorporated host phylogenetic information into the GAMs, using the MRFtools R package. This greatly improved model performance

Table 3: Fitted GAM models of mean parasite body size as a function of host body size (phylogenetic smoothed penalty).

Response	Predictor	edf	Ref.df	F	p-value	R^2
Mean parasite width	Adult mass (g)	4.72	5.39	6.21	0.0001	0.405
	Host species	51.57	358.00	0.57	0.0001	
Mean parasite length	Adult mass (g)	6.24	6.94	17.80	0.0001	0.436
	Host species	37.23	360.00	0.32	0.0001	
Mean parasite volume	Adult mass (g)	8.27	8.74	58.30	0.0001	0.641
	Host species	20.61	363.00	0.12	0.0001	
Mean parasite width	Adult mass (g)	1.00	1.00	0.17	0.7485	1.000
	Host species	279.00	281.00	916698.59	0.0008	
Mean parasite length	Adult mass (g)	1.86	1.94	0.61	0.5782	0.900
	Host species	221.49	279.00	8.96	0.0001	
Mean parasite volume	Adult mass (g)	1.00	1.00	0.33	0.6699	1.000
	Host species	279.00	281.00	1030826.23	0.0008	
Mean parasite width	Adult mass (g)	7.13	7.68	21.57	0.0001	0.858
	Host species	73.27	165.00	2.53	0.0001	
Mean parasite length	Adult mass (g)	1.00	1.00	0.00	0.9760	0.893
	Host species	122.63	165.00	8.43	0.0001	
Mean parasite volume	Adult mass (g)	6.80	7.17	22.98	0.0001	0.919
	Host species	113.79	165.00	7.50	0.0001	

– as estimated using R^2 – but this was almost certainly due to the model overfitting to the host phylogenetic data (Table 3). This is evidenced by the large values of estimated degrees of freedom (edf) for the host species smooth terms as well.

Comparison of GAM results with Spearman's correlations

We also explored the agreement between the fitted GAMs (without phylogenetic correction) and a simple Spearman's correlation coefficient, finding the results largely in agreement (Table 4).

Cross validation of GAM models

Assessing the models capability of predicting body size based on host or parasite body size was tested using cross-validation, in which we divided the data into training (75%) and testing (25%) sets. This tended to decrease model performance (Table 5). This is not surprising, though it does suggest that the R^2 values from the fitted GAM models on the full data must be interpreted cautiously, and certainly not as a measure of potential predictive performance. Despite this, the detected relationships from the main text are still interesting, though they are quite weak. This further strengthens the narrative that provides limited support for Harrison's rule across a variety of host taxa.

Separating out juvenile parasites

We opted to only consider helminth parasites in their adult stage, as larval stages may be far more variable in size. Here, we partition out the larval and adult parasitic stages to compare the GAM model fits when fitting solely to all parasite stages (Table 6) or solely to larval data (Table 7). We find that the GAM models poorly fit the larval parasite data (Table 7). This could be due to limitations in available data, where models were fit to approximately 100 data points, whereas we had at least three times as much data for adult helminth parasites.

Table 4: Fitted generalized additive models for Harrison's rule for both host (top rows) or helminth parasite (bottom rows) perspective provide support for positive host-parasite body size scaling relationships. *df* is degrees of freedom used for F-test, *edf* is the estimated complexity of the smoothed term, and adj. R^2 is the adjusted variance explained. We also report the results of a Spearman's correlation between the two variables to provide additional support for the observed relationships (ρ and p-value).

Host group	Response	Predictor	p-value	adj. R^2	ρ	p-value
Mammals	Mean parasite width	s(host mass)	<**0.001**	0.07	0.249	<**0.001**
	Mean parasite length	s(host mass)	<**0.001**	0.26	0.22	<**0.001**
	Mean parasite biovolume	s(host mass)	<**0.001**	0.60	0.23	<**0.001**
Birds	Mean parasite width	s(host mass)	0.51	0.00	0.30	<**0.001**
	Mean parasite length	s(host mass)	0.35	0.01	0.25	<**0.001**
	Mean parasite biovolume	s(host mass)	0.94	0.00	0.34	<**0.001**
Fish	Mean parasite width	s(host mass)	<**0.001**	0.38	0.30	<**0.001**
	Mean parasite length	s(host mass)	0.85	0.00	0.36	<**0.001**
	Mean parasite biovolume	s(host mass)	<**0.001**	0.21	0.34	<**0.001**
Mammals	Mean host size	s(Width)	**0.01**	0.05	0.13	<**0.001**
	Mean host size	s(Length)	<**0.001**	0.09	0.12	**0.0012**
	Mean host size	s(Biovolume)	<**0.001**	0.38	0.15	**0.0024**
Birds	Mean host size	s(Width)	0.39	0.01	0.01	**0.0005**
	Mean host size	s(Length)	0.16	0.03	0.08	0.87
	Mean host size	s(Biovolume)	<**0.001**	0.17	0.04	0.19
Fish	Mean host size	s(Width)	<**0.001**	0.40	0.27	<**0.001**
	Mean host size	s(Length)	0.90	0.00	0.22	<**0.001**
	Mean host size	s(Biovolume)	<**0.001**	0.15	0.27	<**0.001**

Table 5: Harrison's rule for both host (top rows) or helminth parasite (bottom rows) perspective provide support for positive host-parasite body size scaling relationships. The estimation of R^2 (mean and sd) are from 100 train-test splits, testing model performance on a 25% test set drawn randomly from the data.

Host group	Response	Predictor	mean R^2	sd R^2
Mammals	Mean parasite width	Adult mass (g)	0.016	0.031
	Mean parasite length	Adult mass (g)	0.064	0.140
	Mean parasite volume	Adult mass (g)	0.081	0.199
Birds	Mean parasite width	Adult mass (g)	0.085	0.166
	Mean parasite length	Adult mass (g)	0.000	0.000
	Mean parasite volume	Adult mass (g)	0.006	0.018
Fish	Mean parasite width	Adult mass (g)	0.001	0.003
	Mean parasite length	Adult mass (g)	0.002	0.004
	Mean parasite volume	Adult mass (g)	0.000	0.000
Mammals	Mean host size	Length	0.000	0.001
	Mean host size	Length	0.000	0.000
	Mean host size	Length	0.000	0.000
Birds	Mean host size	Width	0.001	0.002
	Mean host size	Width	0.007	0.014
	Mean host size	Width	0.001	0.003
Fish	Mean host size	Biovolume	0.001	0.007
	Mean host size	Biovolume	0.000	0.001
	Mean host size	Biovolume	0.000	0.004

Table 6: Fitted generalized additive models for Harrison's rule considering all parasite stages (including larval stages) of helminth parasites. *df* is degrees of freedom used for F-test, *edf* is the estimated complexity of the smoothed term, and adj. R^2 is the adjusted variance explained.

Host group	Predictor	edf	df	F	p-value	R^2
Mammals	s(Width)	7.34	8.31	2.58	**0.01**	0.04
	s(Length)	7.79	8.62	5.41	**<0.001**	0.09
	s(Biovolume)	8.46	8.87	28.27	**<0.001**	0.38
Birds	s(Width)	2.07	2.53	1.33	0.38	0.01
	s(Length)	6.26	7.29	1.52	0.15	0.03
	s(Biovolume)	8.04	8.69	6.38	**<0.001**	0.17
Fish	s(Width)	8.52	8.93	14.98	**<0.001**	0.32
	s(Length)	7.23	8.00	1.25	0.27	0.02
	s(Biovolume)	8.53	8.88	4.23	**<0.001**	0.10

Table 7: Fitted generalized additive models for Harrison's rule considering only larval helminth parasite stages. *df* is degrees of freedom used for F-test, *edf* is the estimated complexity of the smoothed term, and adj. R^2 is the adjusted variance explained.

Host group	Predictor	edf	df	F	p-value	R^2
Mammals	s(Width)	1.00	1.00	0.06	0.81	0.00
	s(Length)	1.00	1.00	0.00	0.99	0.00
	s(Biovolume)	1.00	1.00	0.01	0.91	0.00
Birds	s(Width)	4.41	5.22	1.99	0.16	0.36
	s(Length)	1.00	1.00	0.01	0.92	0.00
	s(Biovolume)	1.38	1.63	0.19	0.77	0.00
Fish	s(Width)	1.79	2.21	0.69	0.61	0.00
	s(Length)	1.00	1.00	0.41	0.52	0.00
	s(Biovolume)	1.00	1.00	0.30	0.58	0.00

Null processes could potentially result in Harrison's rule

Here, we provide fitted generalized additive models for the null model distributions of host-parasite body size scaling relationships presented in the main text. We see that even when model coefficients are significantly different from zero, the amount of variance explained by the model is effectively none (Table 8), suggesting that it is unlikely for null processes to generate Harrison's rule.

Table 8: Fitted generalized additive models for Harrison's rule for both host (top three rows) or helminth parasite (bottom three rows) perspective fit to data from the null model provide no support for host-parasite body size scaling relationships. *df* is degrees of freedom used for F-test, *edf* is the estimated complexity of the smoothed term, and adj. R^2 is the adjusted variance explained.

Response	Predictor	edf	df	F	p-value	R^2
Mean parasite width	s(adult mass)	7.63	8.31	3.78	<**0.001**	0.00
Mean parasite length	s(adult mass)	2.00	2.24	6.98	<**0.001**	0.00
Mean parasite biovolume	s(adult mass)	2.60	3.01	3.62	**0.01**	0.00
Mean host size	s(Width)	1.00	1.00	0.36	0.55	0.00
Mean host size	s(Length)	1.00	1.00	0.42	0.52	0.00
Mean host size	s(Biovolume)	1.00	1.00	0.11	0.74	0.00

Upper threshold constraints on parasite size

It is possible that small parasites can infect all host species, but that the maximum size a parasite can be is constrained by host body mass. We explored this by considering the maximum parasite size (in terms of length, width, and biovolume) as it relates to host body size. We found clear positive scaling relationships for maximum parasite size just as we saw for mean host-parasite size scaling relationships (Figure 1 and Table 9) relative a null model (Table 10). Note that fitted models were able to explain less of the variation in maximum parasite size relative to mean parasite size (Table 9).

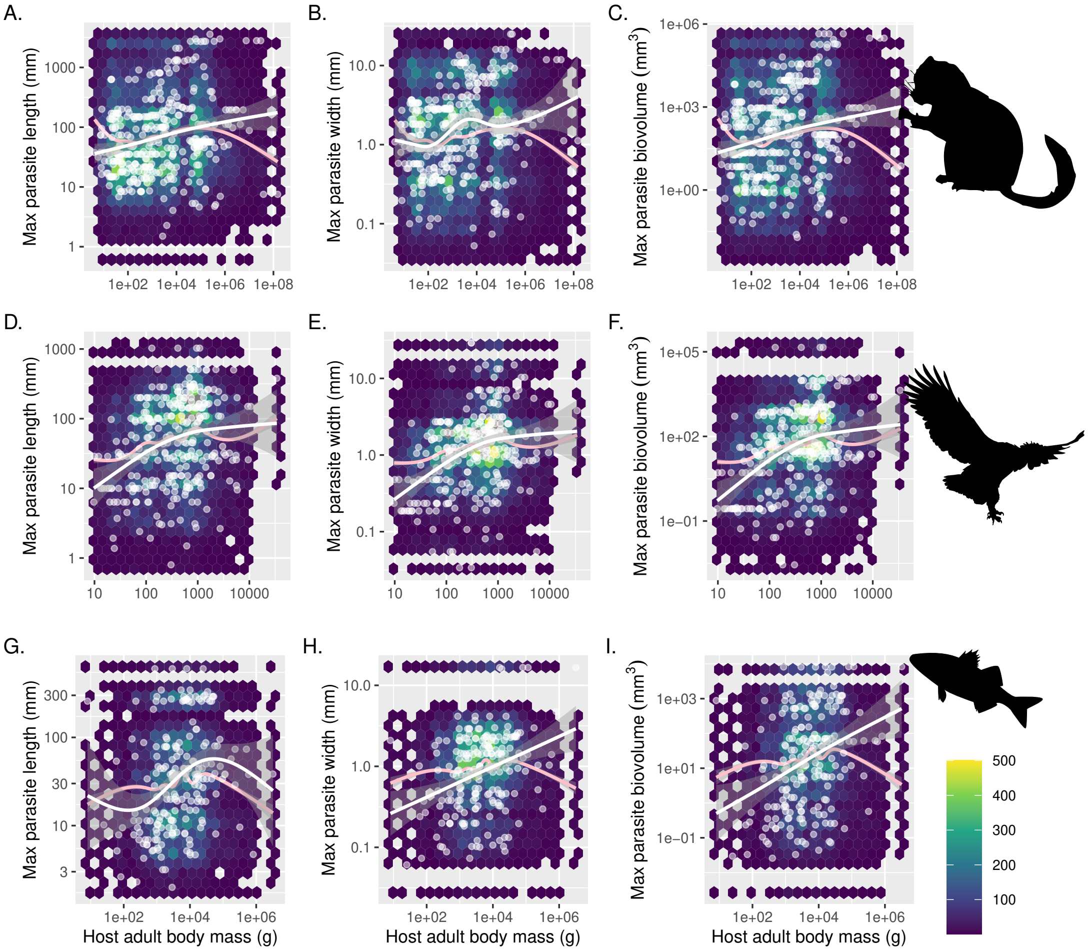

Figure 1: **Evidence for Harrison's rule from the host perspective**. Host body size was positively related to maximum parasite length (**a**), maximum parasite width (**b**), and maximum parasite biovolume (**c**). White points and lines correspond to the empirical data and fits from generalized additive models, while background color gradient and pink lines correspond to the underlying null distribution and generalized additive model fit to null data.

Table 9: The relationship between smoothed host mass and a) maximum parasite width, b) length, and c) biovolume for mammals, birds, and fish. *df* is degrees of freedom used for F-test, *edf* is the estimated complexity of the smoothed term, and adj. R^2 is the adjusted variance explained.

Host group	Response	Predictor	edf	df	F	p-value	adj. R^2
Mammals	Max parasite width	s(adult mass)	3.67	4.29	3.22	**0.01**	0.03
	Max parasite length	s(adult mass)	8.50	8.89	8.22	**<0.001**	0.16
	Max parasite biovolume	s(adult mass)	8.65	8.94	11.23	**<0.001**	0.21
Birds	Max parasite width	s(adult mass)	5.64	6.62	1.91	0.06	0.03
	Max parasite length	s(adult mass)	5.04	5.93	3.79	**<0.001**	0.06
	Max parasite biovolume	s(adult mass)	1.00	1.00	0.03	0.85	0.00
Fish	Max parasite width	s(adult mass)	8.36	8.87	9.79	**<0.001**	0.28
	Max parasite length	s(adult mass)	1.00	1.00	0.27	0.60	0.00
	Max parasite biovolume	s(adult mass)	4.72	5.50	6.49	**<0.001**	0.14

Table 10: Null models relating smoothed host mass to maximum parasite width, length, and biovolume for mammals, birds, and fish. While all coefficients were significant, the variance explained was minimal. *df* is degrees of freedom used for F-test, *edf* is the estimated complexity of the smoothed term, and adj. R^2 is the adjusted variance explained.

Host group	Response	Predictor	edf	df	F	p-value	adj. R^2
Mammals	Max parasite width	s(adult mass)	8.88	8.99	13.16	**<0.001**	0.00
	Max parasite length	s(adult mass)	2.97	3.46	11.14	**<0.001**	0.00
	Max parasite biovolume	s(adult mass)	3.28	3.84	5.13	**<0.001**	0.00
Birds	Max parasite width	s(adult mass)	8.95	9.00	60.51	**<0.001**	0.02
	Max parasite length	s(adult mass)	8.97	9.00	102.08	**<0.001**	0.03
	Max parasite biovolume	s(adult mass)	8.93	9.00	19.62	**<0.001**	0.01
Fish	Max parasite width	s(adult mass)	8.06	8.73	16.82	**<0.001**	0.01
	Max parasite length	s(adult mass)	8.30	8.84	23.17	**<0.001**	0.01
	Max parasite biovolume	s(adult mass)	7.67	8.49	12.89	**<0.001**	0.01

Do larger host species just have more parasites?

It is possible that larger-bodied host species could have larger parasites simply because larger host species also have higher parasite species richness. Previous meta-analyses have clearly linked increased parasite species richness with increasing host body size and geographic range size [5]. While the coefficient from a generalized additive model of parasite species richness as a function of adult host body size was significant (β=2.61, $p < 0.001$), the model explained functionally no variation in the data ($R^2 = 0.00$). Further, when we use 100 null model randomizations per host species, randomly shuffling interactions while constraining parasite species richness to the observed data, we see no difference between the null distribution and the empirical data (Figure 2).

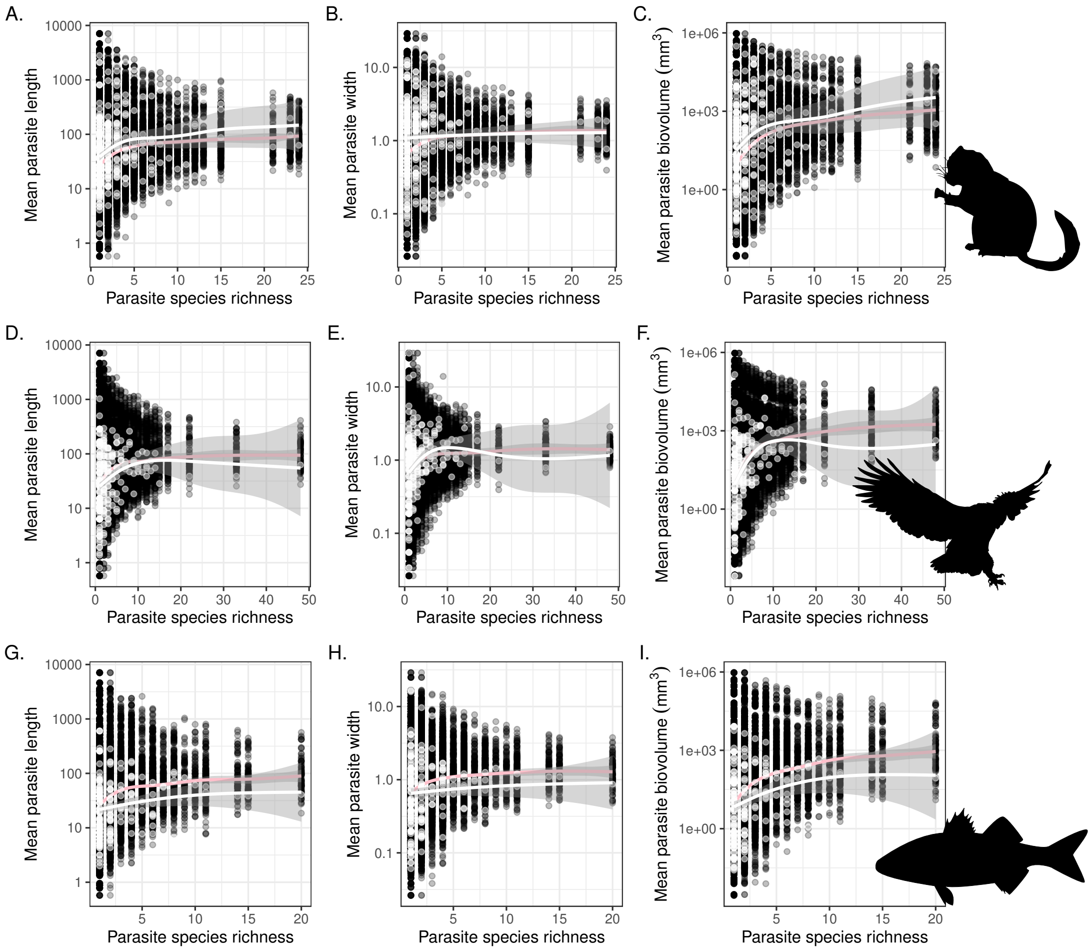

Figure 2: Mean parasite body size, measured as mean parasite length (**a,d,g**), width (**b,e,h**), or biovolume (**c,f,i**), was unrelated to the number of parasite species infecting a host species (i.e., parasite species richness) across mammals, birds, and fish. Black points and the pink line correspond to null model randomizations, while white points and line correspond to the empirical data and the fitted generalized additive model.

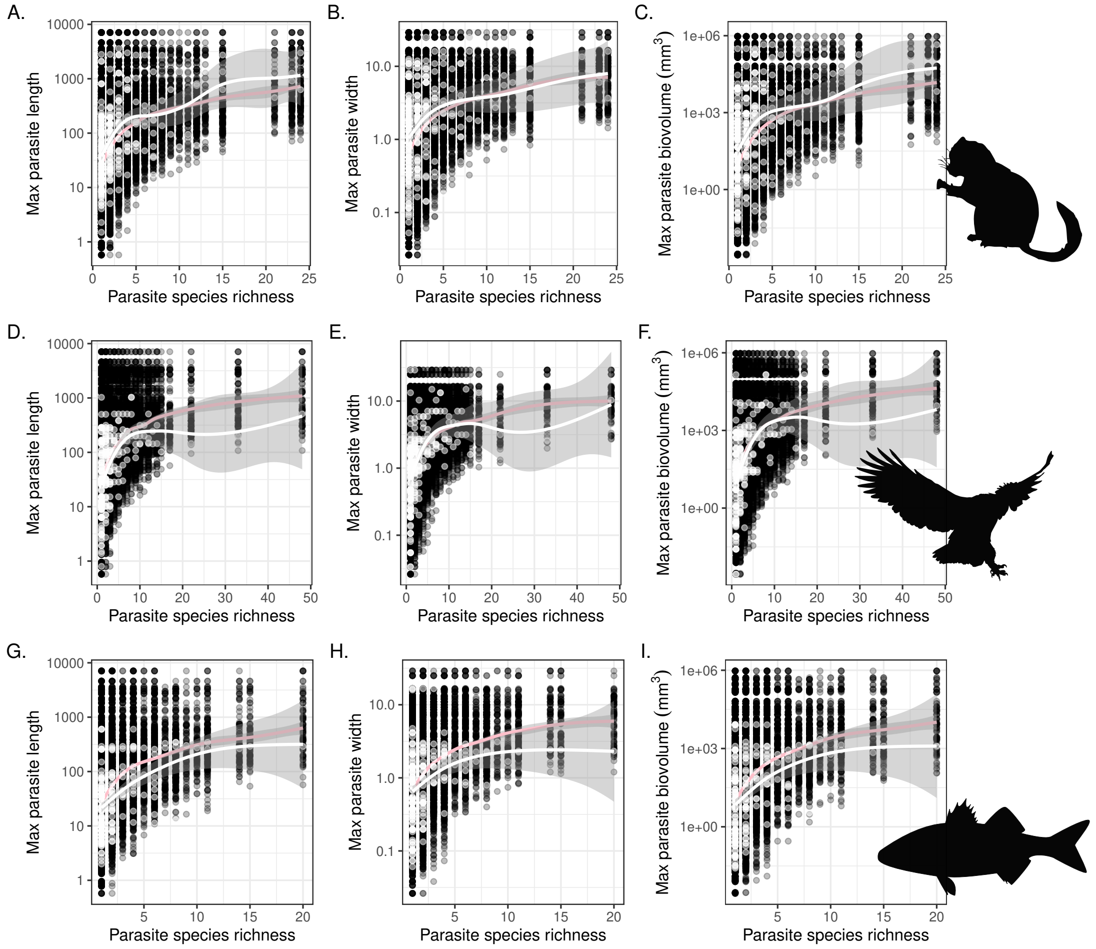

Figure 3: Maximum parasite body size, measured as the maximum observed parasite length (**a,d,g**), width (**b,e,h**), or biovolume (**c,f,i**), generally increased with increasing number of parasite species infecting a host species (i.e., parasite species richness) across mammals, birds, and fish. However, this is largely a statistical artifact, and is expected under our null model (black points and pink lines correspond to null model randomizations). Empirical data correspond to the white points, with the white line being the fitted generalized additive model.

Do smaller parasite species have a broader host range?

Larger parasites may be constrained to only infect larger hosts, but the opposite may not be true (i.e., smaller parasites may be able to infect a wider range of host body sizes). We explored this by considering the relationship between parasite body size (length, width, and biovolume) and parasite host range (measured as the number of host species a parasite infects). We failed to detect any evidence that smaller parasite species were able to infect more host species (Figure 4), instead finding consistent positive (but non-significant) relationships between aspects of parasite body size and host range. That is, larger parasite species tended to have slightly larger host ranges, but these relationships were not statistically significant.

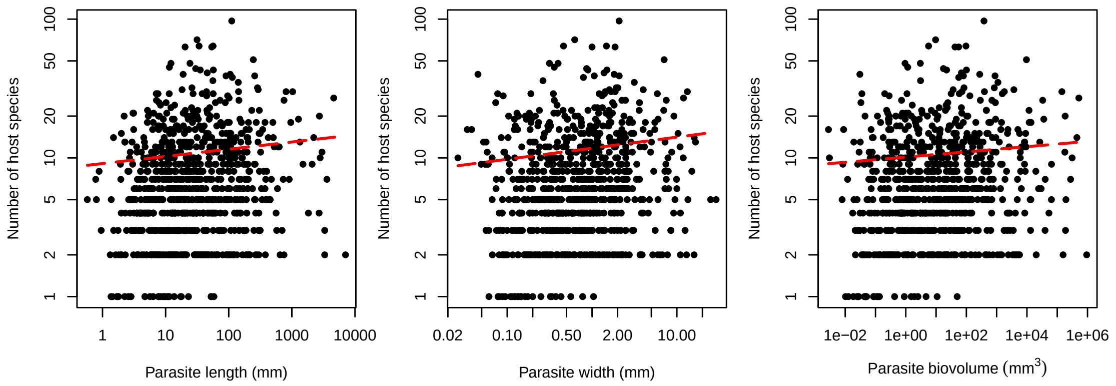

Figure 4: Smaller parasite species do not have a broader host range. Each point is a parasite species, with fitted linear models shown as red dotted lines. None of these linear models results in a statistically significant relationship between parasite body size (length, width, or biovolume) and parasite host range (measured as the number of host species a parasite infects).

References

[1] Upham, N. S., Esselstyn, J. A. & Jetz, W., 2019 Inferring the mammal tree: species-level sets of phylogenies for questions in ecology, evolution, and conservation. *PLoS biology* **17**, e3000494.

[2] Rabosky, D. L., Chang, J., Cowman, P. F., Sallan, L., Friedman, M., Kaschner, K., Garilao, C., Near, T. J., Coll, M., Alfaro, M. E. *et al.*, 2018 An inverse latitudinal gradient in speciation rate for marine fishes. *Nature* **559**, 392–395.

[3] McTavish, E. J., Gerbracht, J. A., Holder, M. T., Iliff, M. J., Lepage, D., Rasmussen, P. C., Redelings, B. D., Sánchez Reyes, L. L. & Miller, E. T., 2025 A complete and dynamic tree of birds. *Proceedings of the National Academy of Sciences* **122**, e2409658122.

[4] Pfenning-Butterworth, A. C., Botero-Cañola, S. & Cressler, C. E., 2021 On the evolution of host specificity: a case study of helminths. *BioRxiv* pp. 2021–02.

[5] Kamiya, T., O'Dwyer, K., Nakagawa, S. & Poulin, R., 2014 What determines species richness of parasitic organisms? a meta-analysis across animal, plant and fungal hosts. *Biological Reviews* **89**, 123–134.
